# Risk Assessment of Electroconvulsive Therapy in Clinical Routine: A 3-Year Analysis of Life-Threatening Events in More Than 3,000 Treatment Sessions

**DOI:** 10.3389/fpsyg.2021.767915

**Published:** 2021-11-23

**Authors:** Vivien L. Hajak, Göran Hajak, Christoph Ziegelmayer, Simone Grimm, Wolfgang Trapp

**Affiliations:** ^1^Department of Psychology, Medical School Berlin, Berlin, Germany; ^2^Department of Psychiatry, Psychosomatic Medicine and Psychotherapy, Social Foundation Bamberg, Bamberg, Germany; ^3^Department of Psychiatry, Charité, Campus Benjamin Franklin, Berlin, Germany; ^4^Department of Psychology, Otto-Friedrich-University of Bamberg, Bamberg, Germany; ^5^Department of Psychology, University of Applied Sciences, Bamberg, Germany

**Keywords:** electroconvulsive therapy, ECT, neurostimulation, depression, live threatening adverse events

## Abstract

**Background:** Extensive research has reported that electroconvulsive therapy (ECT) can be highly effective in approximately 80% of patients suffering from depression. Its clinical use is mainly limited by historical objections and the concern about unwanted adverse effects (AEs), including serious and potentially life-threatening adverse events (pLTAEs), induced either by ECT or by anesthesia. Objective risk estimation is, therefore, a decisive factor in determining an indication for ECT.

**Methods:** This paper presents a retrospective analysis of 3-year safety protocols and patient files of 157 patients who received a total of 3,106 ECT applications in a psychiatric inpatient setting at a psychiatric community hospital. This patient group comprises 5.3% of inpatients admitted with comparable diagnoses. Adverse events were analyzed from standardized safety protocols and patient files with a focus on pLTAEs.

**Results:** Adverse events were reported for 30 (19.1%) of the 157 participants during 39 (6.1%) of 641 hospital stays. Serious pLTAEs occurred during three electroconvulsive stimulations in three patients, who needed action through the administration of medication or mechanical respiration. No patient suffered permanent damage to health, and no patient died. The incidence of these and other AEs was independent of sex, age, and diagnosis of patients, and anesthesia medication. Minor AEs occurred more often with higher stimulus doses and an increasing number of treatments.

**Conclusion:** The low incidence rate of 0.097% of serious pLTAEs that require medical action may allow the conclusion that ECT is a rather safe treatment when performed in a controlled setting. The beneficial risk profile of ECT performed in the standard care of psychiatric hospitals suggests a more generous indication of this treatment method. We recommend that ECT facilities collect individual safety data to allow a reliable judgment of their institutional ECT risk profile.

## Introduction

World Health Organization studies have highlighted psychiatric disorders to be a leading cause of human disability worldwide (World Health Organization [WHO], 2021). Mental and addictive disorders affect more than 1 billion people globally (Rehm and Shield, 2019). The global burden of mental illness accounts for 32.4% of years lived with disability (YLDs) and 13.0% of disability-adjusted life years (DALYs) (Vigo et al., 2016).

A wide armamentarium of treatment options suggests psychiatric disorders to be well-treatable diseases. Treatment techniques range from a variety of psychotherapies and psychotropic medications to behavioral, physical, and socio-therapeutic approaches to neurophysiological procedures. However, acute and severe episodes of schizophrenia, major depressive disorder (MDD), and bipolar depression are treatment resistant (Fekadu et al., 2009; Fornaro et al., 2020; Campana et al., 2021), and a delayed response to medication and psychotherapy remain major issues to treatment. Depressive episodes ranging between 15 and 30% fail to respond to adequate trials of two antidepressants, and 68% of individuals do not achieve remission from depression after a first-line course of antidepressant treatment (Dodd et al., 2020). Modern psychiatry has therefore opened up to invasive and highly technical treatment options. The latter include techniques of neurostimulation, with electroconvulsive therapy (ECT) being the oldest among these and all other biological treatments introduced in psychiatry (Kaliora et al., 2018).

Virtually, all recently published national and international guidelines and meta-analyses on the treatment of depression and schizophrenia mention the above-listed treatments but consider ECT as the most valuable option in case of treatment resistance and severe and life-threatening mental conditions, especially depression and schizophrenia (Bauer et al., 2015; Hasan et al., 2015; DGPPN et al., 2017; Gaebel et al., 2019). Consequently, the vast majority of all ECT treatments are delivered to patients with MDD. Empirical data concerning the therapeutic effects of ECT are impressive. Meta-analyses of ECT for depression and schizophrenia support robust treatment effects with odds ratios between three and four and excellent remission rates of up to 75% even in treatment resistance, improving health-related quality of life (Giacobbe et al., 2018; Bahji et al., 2019; Grover et al., 2019; Mutz et al., 2019; Sinclair et al., 2019; Chen et al., 2021; Li et al., 2021). Independent of its antidepressant effects, ECT also has a significant anti-suicidal effect (Kellner et al., 2005). Therefore, ECT has been recommended as the first choice for the treatment of patients at a high risk of suicide (Fink et al., 2014).

Despite this and other extensive evidence of significant therapeutic effects of ECT in various mental disorders (Gazdag et al., 2009; van Diermen et al., 2018; Grover et al., 2019; Sinclair et al., 2019; Chiu et al., 2020; Kellner et al., 2020; Elias et al., 2021; Yahya and Khawaja, 2021), from children (Døssing and Pagsberg, 2021) to the elderly (Kellner et al., 2016; McCall et al., 2018), both short and long term (Ward et al., 2018), ECT experts judge that ECT is highly underutilized. Indeed, ECT is mainly used as one of the last treatment options for patients with depression and schizophrenia. This is attributed to persistent stigma (Gazdag et al., 2017b; Rzesnitzek and Lang, 2017; Alexander et al., 2020a,b) and the lack of knowledge about modern ECT techniques and their risk-benefit ratio (Grover et al., 2019; Kellner et al., 2020). Physicians, as well as patients, are often hesitant to administer or accept ECT (Birkenhager and van Diermen, 2020), which, at first glance, appears to be dangerous because of its invasive approach. Still, experienced psychiatrists are often unaware of the well-defined standards for ECT and anesthetic procedures that ensure the best possible safety during implementation (American Psychiatric Association, 2001; Waite and Easton, 2013). In line with this position, most treatment guidelines for depression and schizophrenia in Germany (Bschor and Adli, 2008; DGPPN et al., 2017; Gaebel et al., 2019) and worldwide (Hasan et al., 2012, 2015; Bauer et al., 2013, 2015; Milev et al., 2016) list ECT as the last step in treatment algorithms, except for catatonia, stupor and life-threatening treatment-resistant mental disorder, for which early ECT is recommended although ECT is considered differently in guidelines for bipolar disorder (Leiknes et al., 2012; Parker et al., 2017; Malhi et al., 2020). The abovementioned issues may contribute to infrequent use of ECT worldwide (Leiknes et al., 2012) and especially in German psychiatry (Vocke et al., 2015; Timäus et al., 2021). To overcome irrational objections to the use of ECT, valid data and public information about the benefits and risks of ECT are essential. Monitoring for antidepressant-associated adverse effects (AEs) in the treatment of patients with MDD has been repeatedly required by international experts (Dodd et al., 2018). The present study contributes to the objectification of the topic by analyzing the risk profile of ECT when applied in a psychiatric community hospital, which represents a standard care institution among German psychiatric institutions.

## Materials and Methods

The study was designed as a retrospective cohort study to analyze the 3-year safety data and respective patient records of all inpatients who underwent ECT as part of the standard of care regime at a large psychiatric community hospital located in northern Bavaria, Germany. Target variables were serious and potentially life-threatening adverse events (pLTAEs) related to the ECT procedure. Analyses were performed following pertinent laws and regulations and the Helsinki Declaration. Informed consent was obtained from the participants for the publication of their case report included in the manuscript (including all data and images). The study was approved by the Ethics Committee of the Department of Psychology, University of Bamberg.

### Sample

Inpatients of the Clinic for Psychiatry, Psychosomatic Medicine, and Psychotherapy of the Social Foundation Bamberg who underwent ECT in the years from 2018 to 2020 were enrolled in this study. Baseline diagnostic assessment and treatment were done by board-certified senior psychiatrists. Patients were admitted to the Center for Neurostimulation according to individual clinical needs as part of the hospital standard care and under consideration of the German national guidelines for the treatment of depression and schizophrenia (DGPPN et al., 2017; Gaebel et al., 2019). All patients met the International Classification of Diseases, 10th Revision (ICD-10; World Health Organization [WHO], 1993] criteria for a psychiatric disorder. Multimodal psychiatric treatment, including psychopharmacological and psychotherapeutic approaches, was continued during ECT treatment.

Participants for the data analyses were screened for eligibility based on a search of the hospital treatment safety protocol recordings for ECT.

Most of the patients included in this report had more than one hospital stay within the observation period. Thus, in the following text, the term “cases (hospital stays)” is used, whenever statistics are based on the number of hospital stays and not on the number of patients.

### Electroconvulsive Therapy Procedure

The ECT application consisted of intravenous, short-term anesthesia with propofol and muscle relaxation with succinylcholine, while a brief electrical stimulus applied to the brain using external electrodes induced an epileptic seizure over several seconds, which is the core feature of a curative effect. It is typically administered by a team of trained medical professionals that includes a psychiatrist, an anesthesiologist, and a nurse or physician assistant, all of whom assure a safe treatment process (American Psychiatric Association, 2001). Details on the technique and application of ECT, as well as easily understandable explanations of indications and risks, can be found in the internet presentations of the American Psychiatric Association (APA; American Psychiatric Association, 2021; The Johns Hopkins University, 2021), the Mayo Clinic (Mayo Foundation for Medical Education and Research [MFMER], 1998-2021), and the video illustration created by West Virginal University of Medicine [WVU] (2021).

In this study, ECT was performed with a Thymatron-System-IV device using right unilateral or bitemporal brief pulse stimulation with 0.75-ms pulse width and a constant current of 900 mA. ECT was applied in an intermediate care unit of the Center of Neurostimulation of the Department of Psychiatry by board-certified psychiatrists with the experience of an average of 3,000 ECTs, board-certified anesthesiologists, and psychiatric nurses (Ziegelmayer et al., 2017). To achieve the highest possible level of safety, an indication for ECT and the application of the treatment were restricted to highly qualified physicians with the experience of at least 250 supervised treatments and an in-house examination of knowledge and skills before being allowed to perform the procedure themselves.

Stimulus dose was determined using the “half-age” method (Petrides and Fink, 1996; Petrides et al., 2009) with stimulus durations ranging from 4.6 to 7.8 s. The dose was increased as needed during the acute course to assure proper convulsion and was kept constant during the maintenance course. Anesthesia was induced with propofol 1–1.5 mg/kg body weight and combined 282 with the muscle relaxant succinylcholine 0.7–1.0 mg/kg body weight.

The treatment consisted of acute and maintenance therapy. Acute therapy followed a treatment regimen with ECT applied three times per week for a period of 2–6 weeks. Following hospital standards, ECT was applied in an inpatient setting. Maintenance therapy was performed to ensure an effective form of relapse prevention after an acute successful course of treatment (Weiner and Reti, 2017; Ward et al., 2018; Luccarelli et al., 2020). It was also applied in an inpatient setting although it should be noted that ECT in an outpatient setting is an option used elsewhere (Lapid et al., 2018; McCall et al., 2018). Maintenance ECT followed a treatment regimen derived from clinical experience. ECT was applied one time a week with increasing intervals up to one time every 6 weeks, depending on the psychopathological findings of the patient. In the case of clinical stability, ECT was terminated after a treatment interval of 6 weeks. In case of clinical deterioration, ECT was continued, with appropriately shortened intervals if necessary.

### Measures

Data on safety and AEs during ECT and the following hour were obtained as part of the hospital quality assessment procedure and were recorded by experienced psychiatrists and anesthesiologists, both of whom attended the ECT session.

Data collection was based on a structured ECT safety protocol, which included a treatment inclusion checklist, a listing of potential risk confounders such as comorbid diseases, a clinical observation of AEs, an AE checklist, and questionnaire as well as an interview of the patient regarding spontaneous AEs, a performance protocol that described the details of the ECT process, including serious pLTAEs and respective treatment measures, as well as electroencephalography (during ECT), and vital parameters as measured with continuous recording of blood pressure, electrocardiography, and oxygen saturation. The electroconvulsive stimulation was followed by 5 h of clinical monitoring of cardiopulmonary parameters and spontaneous AEs.

Candidates with acute AEs during ECT and in the following 5 h were confirmed and characterized by a detailed analysis of the clinical patient file of the given hospital stay. Patients who experienced pLTAEs underwent a full data analysis, including patient files covering all hospital stays with an application of ECT.

### Statistical Analysis

*T*-tests for independent samples were used to compare the groups with minor side effects and no side effects concerning propofol, succinylcholine, age, and the total number of received sessions. Chi-squared tests were used to analyze whether gender or main diagnosis was associated with the occurrence of minor side effects. Alpha was set to 0.05 for all analyses.

## Results

### Patient Sample

From the beginning of 2018 until the end of 2020, 1,144 patients were referred for inpatient treatment with a diagnosis of recurrent MDD (WHO ICD-10 Code F33.x; World Health Organization [WHO], 1993), 752 were suffering from a single episode of MDD (F32.x), 326 were suffering from bipolar disorder (F31.x), 261 from schizoaffective disorder (F25.x), 481 from schizophrenia (F20.x), and 1 patient from an unspecified mental disorder due to brain damage and dysfunction and to physical disease (F06.9).

During the same period, 157 patients (100 women and 57 men) with a mean age of 55.5 years (*SD* = 15.1 years) were treated with ECT during 641 hospital stays, 406 (63.3%) received ECT during the maintenance course (1 session), and 235 (36.7%) during hospital stays with a series of ECT sessions. In total, this sample received 3,106 ECT sessions.

The largest proportion of this sample had a main diagnosis of a recurrent MDD (F33.x, 59.9%), 7.0% were suffering from a single episode of MDD (F32.x), 12.1% were suffering from bipolar disorder (F31.x), 10.8% from schizoaffective disorders (F25.x), 9.6% from schizophrenia (F20.x), and one participant from an unspecified mental disorder due to brain damage and dysfunction and to physical disease (F06.9). In total, in 27.0% of the patients with hospital stays due to a recurrent MDD (F33.x), 3.2% F32, 20.5% F31, 16.6% F25, 9.7% F20, and 1.1% F06 received ECT treatment.

Table 1 displays the clinical and demographic characteristics of ECT participants, and the total number of inpatients admitted to the hospital with the respective diagnoses.

**TABLE 1:** Sample characteristics.

	Participants		Cases (hospital stays)	
	N	%	N	%
**Gender**				
Male	57	36.3	170	26.5
Female	100	63.7	471	73.5
**Diagnosis**				
F06 unspecified mental disorder	1	0.6	1	0.2
F20 schizophrenia	15	9.6	71	11.1
F25 schizoaffective disorder	17	10.8	64	10.0
F31 bipolar disorder	19	12.1	86	13.4
F32 major depressive disorder	11	7.0	27	4.2
F33 recurrent major depressive disorder	94	59.9	392	61.2
**Adverse events**				
None	124	79.0	599	93.4
Mild	30	19.1	39	6.1
Serious	3	1.9	3	0.5
**Type of adverse event**				
None			599	93.4
Cognitive			11	1.7
Cardiovascular			6	0.9
Cerebral			11	1.7
Muscular			4	0.6
Anesthesia-related			7	1.1
Serious			3	0.5
**Reasons for termination of ECT**				
Not terminated			618	96.4
Mild side effects			9	1.4
Lack of response			8	1.2
Patient request			2	0.3
Other (thrombophlebitis)			1	0.2
Serious adverse event			3	0.5
**Type of ECT treatment**				
Maintenance course (1 session)			406	63.3
Series of ECT sessions			235	36.7
**Type of stimulation**				
Unilateral			619	96.6
Bitemporal			14	2.2
Mixed unilateral and bitemporal			8	1.2

### Stimulation Procedure

Most ECTs were administered using unilateral pulse stimulation (96.6%). A smaller proportion of the sample was either stimulated bitemporally (2.2%) or received a mixture of unilateral and bitemporal stimulations across the individual sessions per stay.

### Group Results

An indication for ECT was made in 8.2% of the patients with recurrent MDD (8.2%), 1.5% of the patients with a single episode of MDD, 3.1% of the patients with schizophrenia, 5.8% of the patients with bipolar disorder, and 6.5% of the patients with a schizoaffective disorder. This patient group compromises 5.3% of inpatients admitted with comparable diagnoses.

**FIGURE 1:**
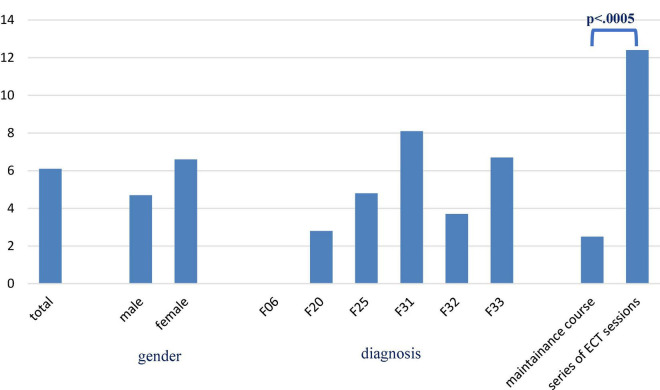
Percentage of patients experiencing mild adverse events (*n* = 641 cases = hospital stays).

In total, 22 of 641 ECT treatments (= hospital stays, 3.4%) were terminated either because of pLTAEs (3 cases, 0.5%), temporary minor side effects (9 cases, 1.4%), the lack of response (8 cases, 1.2%), the request of the patients (2 cases, 0.3%), and other reasons (1 case of thrombophlebitis, 0.2%). Temporary minor side effects were reported in 30 (19.1%) of the 157 participants during 39 (6.1%) of the 641 hospital stays. Eleven cases of cognitive, 6 cases of cardiovascular, 11 cases of cerebral, 11 cases of muscular, and 7 cases of anesthesia-related side effects were reported (see Table 1). Neither age, propofol nor succinylcholine dose was associated with these minor side effects. However, a higher proportion of minor temporary side effects were found for participants with higher stimulus doses and a higher number of ECT sessions (refer to Table 2 and Figure 1). In three cases, pLTAEs occurred (refer to the detailed information for each of these cases mentioned below). In two of these three patients, these pLTAEs lead to ECT termination. All pLTAEs and minor side effects occurred under unilateral stimulation.

**TABLE 2:** Mild adverse events, characteristics of patients, and electroconvulsive therapy (ECT) parameters.

	Participants		Significance	
	N	%	c^2^	*p*
**Gender**				
Male	8	4.7	0.762	0.383
Female	31	6.6		
**Diagnosis**				
F06	0	0.0	2.706	0.745
F20	2	2.8		
F25	3	4.8		
F31	7	8.1		
F32	1	3.7		
F33	26	6.7		
**Type of ECT treatment**				
Maintenance course (1 session)	10	2.5	26.654	<0.0001
Series of ECT sessions	29	12.4		

	**Mean**	**Std**	***T***	***p***

**Age**				
Total			1.669	0.096
No adverse events (*n* = 599)	56.47	15.03		
Mild adverse events (*n* = 39)	60.62	15.19		
**Anesthesia (mg propofol)**				
Total	124.70	29.65	1.149	0.251
No adverse events (*n* = 599)	125.03	29.73		
Mild adverse events (*n* = 39)	119.39	28.74		
**Muscle relaxant (mg succinylcholine)**				
Total	44.06	10.69	0.071	0.943
No adverse events (*n* = 599)	44.05	10.65		
Mild adverse events (*n* = 39)	43.93	11.70		
**Stimulus dose (mA)**				
Total	0.53	0.13	2.011	0.045
No adverse events (*n* = 599)	0.52	0.13		
Mild adverse events (*n* = 39)	0.57	0.12		
**Sessions per stay**				
Total	4.87	6.63	2.683	0.007
No adverse events (*n* = 599)	4.71	6.58		
Mild adverse events (*n* = 39)	7.64	7.05		

### Cases With Potentially Life-Threatening Adverse Events

Three patients experienced an AE that required immediate therapeutic intervention by a doctor and led to the termination of ECT. The cases are presented in the light of the question of which pLTAEs have been caused by ECT, thereby not considering guidelines for presenting a complete patient case and story (Riley et al., 2017). The diagnostic assignment was carried out according to the 10th Revision of the WHO’s; International Classification of Diseases (ICD-10; World Health Organization [WHO], 1993), which has been the current standard instrument for disease classification in Germany.

#### Case 1, Female Patient With Schizoaffective Disorder, 57 Years of Age

A 57-year-old woman (70 kg body weight) with a schizoaffective disorder, currently in a mixed state (ICD-10 F25.2; World Health Organization [WHO], 1993), was scheduled for her first ECT. She had been suffering from reoccurring manic and depressive episodes combined with auditory hallucinations and delusions for more than 30 years. She had been a psychiatric inpatient for a total of nine times with up to 5 months duration and several weeks being treated in closed wards due to suicidal ideation and threats toward other people.

The present episode showed an excited and restless woman with intense energy and racing thoughts in a mixed emotional state, the latter determined by auditory hallucinations, like hearing the voice of her father and the devil as well as delusion, i.e., communication with Jesus and his angels. She also felt that people were talking about her and her role as supernatural as a hope for the liberation of mankind. Inpatient admission for ECT was carried out by a psychiatrist with the consent of the patient. Both hoped to overcome the treatment-resistant disorder, shorten the usually long hospital stays, and reduce the antipsychotic medication that caused extrapyramidal symptoms and weight gain.

Her physical condition was unimpaired, the internal and neurological examination revealed no pathological findings, nor did standard laboratory testing, chest x-ray, electroencephalography, electrocardiography, and cranial CT. Medication before starting ECT included aripiprazole long-acting injectable 400 mg intramuscular one time a month, olanzapine 20 mg/day, biperiden hydrochloride 2 mg/day, mirtazapine 15 mg/day, valproate 1,500 mg/day, lamotrigine 150 mg/day, and lorazepam up to 1.5 mg/day.

Electroconvulsive therapy was performed after the written informed consent in an intermediate care ward with cardiopulmonary recording in the presence of a psychiatrist, an anesthetist, and a psychiatric nurse. A total of 100 mg of propofol and 40 mg of succinylcholine were used for general anesthesia. Stimulation was applied unilaterally on the right side with an energy of 55%. ECT caused a seizure lasting 21 s. The stimulation procedure ended 9 min after the initiation of anesthesia. An extended muscle relaxation followed with altered ability to breathe properly and respiratory acidosis with a pH value of 7.277 (normal value 7.370–7.450) and partial pressure of carbon dioxide (pCO_2_) of 134 mm of mercury (mmHg) (normal value 71–104 mmHg). Sedation with propofol and midazolam was continued, ventilation was carried out using a ring mask over 19 min, and oxygen insufflation was applied. Anesthesia ended 19 min after the end of ECT, oxygen application was stopped 29 min later. The patient was then wide awake with sufficient spontaneous respiratory function.

Subsequent laboratory testing showed a reduction of cholinesterase (CHE) in the serum to 4,215 units/L in comparison to normal levels between 5,320 and 12,920 units/L. CHE-inhibition through dibucaine was 56 with > 70% inhibition, indicating a normal CHE variant, 30–70% inhibition, suggesting a heterozygote atypical CHE variant, and < 30% inhibition being typical for a homozygote atypical CHE variant.

Consequently, ECT treatment was discontinued from the therapy plan due to the abnormality of the CHE system with an increased risk for anesthesia.

#### Case 2, Male Patient With Major Depressive Disorder, 75 Years of Age

The 75-year-old man (93 kg body weight) was an experienced ECT patient who had received two series of ECT at age 65 years for recurrent and severe MDD without psychotic features (ICD-10 F33.2; World Health Organization [WHO], 1993). The ECTs have always been well-tolerated, including one series of maintenance treatments over 6 months. The current depressive episode lasted to 5 months when the patient was referred to inpatient treatment and was characterized by pervasive low mood, loss of interest, lack of energy, and anhedonia. The patient complained of deep sadness with an inability to cry and severe difficulty of concentration and attention and reported no perspective or hope of improvement. Psychiatric outpatient treatment found him to be difficult to treat with around 30% response to several different psychotropic drugs, leaving the patient still deeply depressed.

His physical condition was impaired by disabling difficulty initiating and maintaining sleep, lack of appetite with weight loss of 10 kg within 5 months, obstipation, difficulty urinating, and lumbar spine pain that has been around for years. Laboratory testing revealed no significant deviation from normal findings, Electrocardiography showed a first-degree AV block, and in the cranial CT, slight global brain atrophy was found, and chest x-ray and electroencephalography were normal. Medication before starting ECT included mirtazapine 30 mg/day, duloxetine 30 mg/day, and tamsulosin hydrochloride 0.4 mg/day.

Electroconvulsive therapy was performed following the patient’s explicit request and after written informed consent in the neurostimulation intermediate care ward using the above mentioned (a.m.) standard setting. A total of 140 mg of propofol and 50 mg of succinylcholine were used for general anesthesia. Electroconvulsive stimulation was applied unilaterally on the right side with an energy of 60%. ECT caused a seizure that did not end after 120 s. Intravenous administration of 2 mg of lorazepam ended the seizure about 7 s after application. From this point on cardiopulmonary monitoring, hypertensive blood pressure readings going up to 210 mmHg were revealed, which were normalized after intravenous treatment with 10 mg of urapidil. After awakening, the patient was disoriented, restless, physically stable after 2 h, but needed orientation assistance during the whole day. Electroencephalography with hyperventilation and flicker provocation was performed two times and 2 and 5 days after the event and showed normal findings.

In summary, this was an unintended prolonged seizure that required pharmaceutical inference of the doctor applying ECT to avoid brain damage and needed the anesthetist to cope with the following hypertensive crisis. ECT was discontinued from the therapy plan due to persisting risk for prolonged seizures.

#### Case 3, Female Patient With Recurrent Severe Major Depressive Disorder With Psychotic Features, 23 Years of Age

The young female patient, with significant obesity and 135 kg body weight at a height of 175 cm, was suffering from recurrent severe MDD with psychotic features (ICD-10 F33.3; World Health Organization [WHO], 1993). Five depressive episodes had occurred since the age of 18 years, the last two of which did not respond well to antidepressive medication. ECT was indicated due to treatment-resistant depression. The patient responded moderately with her symptomatology after receiving an ECT series with 17 treatments which she tolerated well. An ECT follow-up included two single ECTs and 1 and 2 weeks after the end of the treatment series. The patient was again admitted to the hospital for maintenance therapy 3 weeks later. Psychopathology was characterized by moderately decreased mood and a reduced feeling of joy but a major lack of drive, which had a significant negative impact on social functioning.

Her physical condition was impaired by obesity and type II diabetes, the latter being treated with oral antidiabetic medication and food restriction. Laboratory testing revealed increased blood sugar and increased triglycerides, while cranial computed tomography, electrocardiography, chest x-ray, and electroencephalography were normal. Medication during maintenance ECT was venlafaxine 225 mg/day, aripiprazole 20 mg/day, and metformin 500 mg/day.

The patient received ECT after written informed consent in the neurostimulation intermediate care ward using the a.m. standard setting. A total of 150 mg of propofol and 50 mg of succinylcholine were used for general anesthesia. ECT was applied unilaterally on the right side at 25% energy. ECT caused a seizure that did not end after 140 s. Intravenous administration of 2 mg of lorazepam ended the seizure in approximately 5 s. From this point on, the continuous cardiopulmonary monitoring revealed normal readings. After awakening, the patient was oriented, and psychomotor functions were slowed down for 20 min, with no pathological findings in her neurological examination. She reported a headache, which disappeared after 2 h. Electroencephalography performed 3 h later showed normal findings.

After 24 h of monitoring, the patient who went home reported a slight improvement in drive and energy and no unwanted side effects. The next ECT was scheduled 4 weeks later and performed with a reduced stimulation energy of 20% and went well, such as the following maintenance ECTs after 5 and 6 weeks.

In summary, this patient, experienced a prolonged seizure, which required pharmaceutical inference, was ended after the pharmacological intervention and did not have prolonged unwanted effects.

## Discussion

The primary aim of this retrospective safety protocol and patient record analysis was to assess acute, serious, and pLTAEs of ECT in the full sample of patients who received ECT as part of the standard of care of a multimodal psychiatric inpatient treatment approach. This was done to assess the risk profile of ECT in clinical practice, to collect facility-specific safety data for appropriate patient education, and to control for operational standards, which assure the survival and prevent physical harm for every single patient willing to receive ECT.

With only 3 pLTAEs occurring during 3 out of 3,106 ECTs applied to 157 patients during 641 hospital stays, ECT performed in the proven facility appears to be a rather safe treatment method. The medical need for action through the administration of medication, particularly, transient mechanical respiration was necessary for all the three affected patients but was part of well-known medical procedures. No patient suffered permanent damage to health, and no patient died. The incidence of AEs was independent of sex, age, and diagnosis of patients, anesthesia medication, and stimulation parameters.

These results should contribute to a better understanding of the risk profile of ECT as a treatment method of choice in psychiatry. We are, however, aware of the fact that a proper indication for ECT by two experienced psychiatrists and carrying out the treatment by an experienced team may have positively influenced the results. Other clinics may achieve completely different results due to different team structures, limited experience, different titration and stimulation protocols, and the use of other medications for anesthesia.

### Indication Rate for Electroconvulsive Therapy

These data were collected in a psychiatric hospital with a statewide well-known Center for Neuro-Stimulation in Psychiatry. Despite the significant positioning of this facility in the clinic, the in-house indication rate for ECT of 5.3% of patients was low in comparison to the number of patients who were referred to the hospital for the treatment of severe depression and other diseases. Most patients received standard multimodal treatment, which was consisted of psychopharmacology, psychotherapy, and adjunctive treatment methods. ECT appeared to be underutilized even in this specialized clinic as it has been reported for some other German psychiatric hospitals with even lower indication rates of 1.72% of all cases with affective disorders and 1.48% of patients with major depression (Timäus et al., 2021). In an older, country-by-country estimation of ECT use, Germany ranged among the countries with the lowest ECT treatment rates worldwide and in Europe. Germany showed a worldwide-treated person rate (TRP) of 0.26, which is the number of ECTs per 10,000 people of the resident population per year. In Europe, only Poland had a lower TRP of 0.11, and Norway and Belgium showed the highest with up to 4.3, respectively, 4.6. TRP of the US Medicare population was 5.1, and up to 4.44 in Australia (Leiknes et al., 2012). Low indications rates for ECT have been confirmed by other surveys in the United States (Wilkinson et al., 2018, 2021) as the reported small group of 0.25% of patients with a mood disorder, privately insured, that received ECT (Wilkinson et al., 2018). A recent meta-analysis produced a composite event rate of 17 people receiving ECT per 100,000 inhabitants in 12 countries, with very high variability between countries. The year of publication was negatively correlated with ECT rates, suggesting a decrease in ECT utilization across time (Lesage et al., 2016).

In Germany, the case-based data of > 1,000,000 cases collected according to §21 of the German hospital remuneration law over 3 years found ECT to be used in 1.72% of all cases with affective disorders and 1.48% with major depressions. Age ≥ 65 years, women, severe and psychotic depression were significantly associated with a higher rate of ECT cases. Greater than 40% of all ECT cases were possibly maintenance ECT cases. The study cohort comprised approximately 35–40% of the annual psychiatric cases and hospitals in Germany and confirmed a rather low treatment rate for depressive inpatients (Timäus et al., 2021). At a first glance, these data contradict a survey among German psychiatrists who agreed that ECT is used less often than it should be to best serve patients’ interests (61–89%) and that it should be applied more often (54–79%). Most of the participants thought that the attitude of ECT has improved among psychiatrists (61–74%) but has not changed among fellow physicians, patients, and the general population (Vocke et al., 2015).

The high variance in ECT use between countries and regions worldwide (Lesage et al., 2016) underlines the importance of data collection in each medical facility that is willing to treat patients with ECT. The data obtained there may help to stimulate referrals for ECT to a certain facility more effectively than reviewing the literature about ECT.

### Electroconvulsive Therapy-Related Potentially Life-Threatening Adverse Events

The authors of this study reported here are aware of the relatively small size of their study, compared to larger observational studies on AEs of ECT and respective reviews that have been published. They present a wide variety of AEs, some of which can cause significant harm to patients. These AEs may be seizure-related, such as prolonged and tardive seizures and status epilepticus, AEs due to muscle relaxants such as prolonged apnea, malignant hypothermia, hyperkalemia, and awareness of neuromuscular paralysis, cardiovascular AEs, such as asystole, raised blood pressure, myocardial infarction, and cardiomyopathy, as well as cognitive AEs such as amnesia and memory loss (Andrade et al., 2016). Regarding the widespread experience of disabled cognition after ECT, it has been proven extensively that ECT may cause cognitive dysfunction but that this condition is reversible and not life-threatening (Semkovska and McLoughlin, 2010; Ziegelmayer et al., 2017; Landry et al., 2021), and that cognitive enhancer may reduce ECT-induced cognitive side effects (Niu et al., 2020). Meta-analyses suggest that new learning is impaired immediately following ECT but that group means to return at least to baseline by 14 days after ECT. Other cognitive functions are generally unaffected. However, the finding of a mean score that is not reduced from baseline cannot be taken to indicate that impairment, particularly of new learning, cannot occur in individuals (Porter et al., 2020). Therefore, monitoring is important as balanced education of patients before treatment. A population-based cohort study in Canada found a total medical event rate of 9.1 (up to 7 days post-treatment), respectively, 16.8 (up to 30 days post-treatment) per 10,000 ECTs, with falls and pneumonia to be the most common events (Blumberger et al., 2017). These data and an extremely low incidence rate of 0.097%, equivalent to approximately one in 1,000 treatments of pLTAEs that required immediate medical action in the data set reported here may allow the conclusion that ECT is a rather safe treatment when performed in a controlled setting.

Undoubtedly, ECT-caused acute death is by far the most frightening AE for patients and physicians and stimulated the authors to perform the study presented here. No patient died during and after 3,106 ECTs performed in 3 years. This finding is in line with low medical morbidity and mortality after ECT in state-based, respective nationwide population-based surveys. Those studies revealed a death rate of 2.4 per 100,000 treatments within 1 day of ECT in Texas (Dennis et al., 2017), mortality rates of less than 0.4 (day of ECT), 1.0 (up to 7 days post-treatment), respectively, 2.4 (up to 30 days post-treatment) per 10,000 ECTs in Canada, and less than 1 death per 73,440 treatments among patients treated in the US Veterans Affairs Healthcare System (Watts et al., 2011). A meta-analysis found all-cause mortality to be 0.42 deaths per 1,000 patients and 0.06 deaths per 1,000 ECTs. Cardiac death accounted for 29% of deaths (Duma et al., 2019). A recent pooled analysis from 15 studies with data from 32 countries estimated ECT-related mortality at 2.1 per 100,000 treatments. A mortality rate of 1.27 of ECT patients vs. 1.94 in Non-ECT controls in a 17-year study in Taiwan suggests that ECT *per se* does not necessarily account for mortality (Liang et al., 2017). A register-based study from Denmark found that the patients who had received ECT had a lower overall mortality rate from natural causes but a slightly higher suicide rate, especially within the first 7 days after the last ECT treatment. This finding underscores the complex task of studying the risk profile of ECT (Munk-Olsen et al., 2007). All the a.m. authors confirmed that death caused by ECT is an extremely rare event (Tørring et al., 2017) and that ECT is a low-risk medical procedure (Blumberger et al., 2017). One could carefully conclude from those data that a physician would have to perform at least 20,000, but more probably 50,000 ECTs to see one death. ECT centers with around 1,000 ECTs per year, like the one in this study, have a negligible statistical probability to see one patient dying due to their ECT.

However, the origin of some studies from outside Europe limits their value for western countries and even more for individual ECT centers, including those in Germany. An indication for ECT may differ from center to center. Referral rates and reasons for ECT as indicated by psychiatrists in hospitals and outpatient practice have never been assessed but appear to be heterogeneous, including the evaluation of comorbid risk factors. Specific expertise and clinical experience of ECT applying staff may vary, and standards of ECT procedures are not uniform. This again suggests that each facility performing ECT may profit from the individually assessed safety data in their institution.

Despite the low risk for serious AEs in statistical analyses, one could learn from each patient how to handle critical situations during ECT. In summary, all patients in this safety protocol analysis had manageable medical problems when receiving ECT, but the cases with a need for medical action may help to better perform ECT in the future.

### Case Report 1

One patient, the 57-year-old lady with schizoaffective disorder (see under results *Case 1*) experienced prolonged paralysis of her muscles during anesthesia due to abnormal CHE function. This was the most remarkable but rarely occurring finding among all ECT patients reported here. Butyrylcholinesterase, also called CHE, is an enzyme synthesized in the liver, which metabolizes neuromuscular blocking agents that are used in anesthesia, such as succinylcholine (Andersson et al., 2019). The latter or other muscle relaxants are essential during ECT to avoid unwanted muscle cramps induced by the seizure. Just one arm is kept without relaxation by a pneumatic cuff to visualize the ECT effect by an increase in muscle tone. The action of succinylcholine is short in onset and is used during a rapid induction of anesthesia, which at a dose of 1 mg/kg body weight is about 45 s, with 10–15 min duration (Andersson et al., 2019). This has to be seen in the light that wide variations in succinylcholine efficacy during ECT anesthesia may require a dose adjustment of 2 SDs either above or below the mean standard dose of around 1 mg/kg body weight (Bryson et al., 2018).

Neuromuscular blockade can be affected by mutations in the CHE gene, where succinylcholine neuromuscular blockade is prolonged (5–10 min) by heterozygosity, while homozygosity may prolong blockade for several hours (Andersson et al., 2019). This butyrylcholinesterase deficiency may go undiagnosed for decades until succinylcholine is used. Studies in Europe estimate a 4% prevalence of congenital serum CHE deficiency in the population (Rosenman and Guss, 1997). The neuromuscular blockade results in extended muscle relaxation and thereby impairs respiratory function after anesthesia as used for ECT (Mollerup and Gätke, 2011). The pathological dibucaine inhibition test in the patient reported here suggested that the adverse drug effect had a pharmacogenetic basis (Kaufman et al., 2011). The value of inhibition, as well as moderate prolongation of muscle relaxation, suggests heterozygosity being present in the given patient. As a result, the rapid action of an anesthetist to prolong sedation and start manual respiration was required to prevent physical harm to the patient.

In summary, this event of prolonged neuromuscular blockade was not related to ECT *per se* but was life-threatening by affecting breathing functioning. This required proper management by an experienced anesthetist. The event may stimulate the discussion about optimizing adjunctive medication regimes in ECT anesthesia, where no consensus exists regarding the optimal anesthetic and muscle relaxing drugs (Soehle and Bochem, 2018).

### Case Report 2

The second patient, the 75-year-old man with severe depression (see under results *Case 2*), experienced a prolonged seizure, which was stopped by intravenous application of benzodiazepine. It is common sense among psychiatrists to limit ECT-induced seizure; however, there is a debate on after which duration a prolonged seizure should be terminated.

Fears of prolonged seizures induced by ECT are manyfold, which are found in doctors more than patients. While the fear to end up in a status epilepticus ranks high, it is reported to occur mainly for single cases (Scott and Riddle, 1989; Whittaker et al., 2007; Dersch et al., 2011). Prolonged seizure is found more often to be 1–2% in the clinical routine where termination by medication runs smoothly (Rasimas et al., 2007). Analyses of ECTs in clinical routine found that women have longer seizures but only at the first treatment session, a strong inverse correlation between age and seizure duration, and a drop in seizure duration along a course of treatments (Rasimas et al., 2007; Hossain and Sullivan, 2008).

Given that many psychotropic medications are thought to either promote or prevent seizures, there is an ongoing concern about concurrent psychotropic medication and ECT administration. In cohort studies, benzodiazepines, antiepileptic agents, selective serotonin-reuptake inhibitors, tricyclic and tetracyclic antidepressants, and stimulants were not associated with seizure threshold or duration, indicating that psychotropic medications may have little effect on seizure at ECT initiation (Chiao et al., 2020). Bupropion showed contradictory results of unimpaired (Chiao et al., 2020) or reduced seizure duration (Takala et al., 2017) but also to cause partial status epilepticus (Dersch et al., 2011). Several other medications increase the duration of seizures, such as caffeine (Bozymski et al., 2018), antibiotics (Kisa et al., 2005), or remifentanil (Takekita et al., 2016). There was no uncontrollable risk with these substances.

In clinical practice, suddenly occurring prolonged seizures can never be ruled out particularly in treatment-resistant patients with combinations of psychotropic drugs (Rucker and Cook, 2008). This, however, could be ruled out widely for our patients who received moderate doses of mirtazapine and duloxetine only. His longer seizure length and several hours period to restore normal consciousness is typically more often occurring with high stimulus intensity. High stimulus intensity is among one of the other factors associated with prolonged reorientation time following seizure as well as an increased risk of post-ECT delirium (Tsujii et al., 2019). Benzodiazepine application was necessary to stop the seizure, and it should be considered that benzodiazepines such as lorazepam have been shown to induce prolonged apnea after ECT-induced prolonged seizure. This negative respiratory effect appears to be rare (Dhavale et al., 2011) and occurs primarily in persons who are acetylcholine (ACH)-deficient and exhibit increased succinylcholine efficacy as described earlier.

The take-home message is that medical termination will be required for patients who could always exhibit a prolonged seizure induced by ECT, which may induce prolonged apnea, and clinicians should be prepared for this contingency.

### Case Report 3

The third patient, a 23-year-old woman with recurrent severe depression (see under results *Case 3*) experienced a prolonged seizure, which was stopped by intravenous application of benzodiazepine. This event mirrored the AEs, which required medical action by a doctor as reported in Case 2.

The young lady was a patient with an unusually high health risk for her age because of being obese and suffering from diabetes. Epidemiological studies on the relationship between diabetes and epilepsy reached discordant conclusions. However, it has been reported that metabolic abnormalities, such as hypoglycemia and hyperglycemia, may increase the prevalence of epilepsy (Marcovecchio et al., 2015). In this line, there are data-driven considerations that mechanisms for the comorbidity of obesity and epilepsy are mitochondrial dysfunction and adiponectin deficiency, which promote epilepsy, obesity, and type II diabetes mellitus (Shlobin and Sander, 2020). While fundamental mechanisms of the relationship between diabetes and epilepsy remain a matter of discussion, hypoglycemia is an accepted reason for the occurrence of seizures in young patients with epilepsy (de Sousa et al., 2020). The ECT patient reported here showed elevated blood sugar levels. However, a decline in the blood sugar level during the previous night and due to the ban of breakfast before ECT, which was applied in the early morning hours, could not be ruled out. While the occurrence of a prolonged seizure after the reduction of blood and brain sugar glucose level would be a possible explanation for our patient’s AE, its probability appears to be low. Epileptic seizures with changes in the blood sugar level are rare and not as common as previously assumed and require significant hypoglycemia to occur (Halawa et al., 2015).

The patient did not show any changes in her respiratory function during ECT despite being overweight. Her obesity might have been a risk factor for altered respiration, in particular when being related to upper airway obstruction and sleep apnea syndrome (SAS; Liu et al., 2017). SAS is well known to increase the risk of anesthesia-related AEs (Opperer et al., 2016; Chan et al., 2019), in particular when spontaneous breathing is needed during intravenous anesthesia. While being obese, no sleep-related respiratory disorder was present in this patient. Nevertheless, her case may remember doctors to consider SAS when examining a patient for ECT, and to use ambulatory screening instruments (Jonas et al., 2017; Rosa et al., 2018) to assess SAS if necessary.

### Electroconvulsive Therapy in the Context of Its Efficacy

It is common sense among physicians that the significance of ECT must be assessed in the context of its safety but also its therapeutic effectiveness compared with other neurostimulation techniques. The latter include repetitive transcranial magnetic stimulation (rTMS), vagus nerve stimulation (VNS), transcranial direct current stimulation (tDCS), deep brain stimulation (DBS), and magnetic seizure therapy (MST). All of them have shown to be effective treatment approaches, rTMS (Brunoni et al., 2017; Sonmez et al., 2019; De Risio et al., 2020), VNS (Bottomley et al., 2019), and tDCS (Fregni et al., 2021) with consistent effects but moderate effect sizes, DBS (Hitti et al., 2020) and MST (Weissman et al., 2020) with promising and positive results in rather small treatment groups, and VNS and DBS being invasive techniques, which require a neurosurgical procedure. Network meta-analytic estimates of non-surgical brain stimulation revealed ECT as the by far most effective treatment for depression with a bitemporal ECT odds ratio of 8.91, high dose right unilateral ECT 7.27, and lower effect sizes of priming rTMS 6.02, MST 5.55, bilateral rTMS 4.92, bilateral theta burst stimulation 4.44, low-frequency right rTMS 3.65, intermittent theta burst stimulation 3.20, high-frequency left rTMS 3.17, and tDCS 2.65 (Mutz et al., 2019). In the comparison of ECT with the widely used technique of rTMS, the latter was better tolerated, while ECT was much more efficacious and did not show a significant signal regarding uncontrollable life-threatening events. The meta-analysis showed cumulative probabilities of being the most efficacious treatment for ECT of 65% while TMS varied between 2 and 25% depending on the type of stimulation (Chan et al., 2019).

These remarkable efficacy data of ECT were mainly found in the predominant indication of therapy-resistant depression and have stimulated ECT to be used successfully in a variety of mental and neurological disorders such as first-episode schizophrenia (Grover et al., 2019), acute mania (Zhang et al., 2021), during the early stages of antidepressive treatment (Haq et al., 2015; van Diermen et al., 2018), for motor and behavioral symptoms of Parkinson’s disease (Takamiya et al., 2021), and for sustaining mood improvement in geriatric depression (Kellner et al., 2016). Furthermore, ECT is particularly effective in patients with depression, including psychotic features and elderly people with depression (van Diermen et al., 2018), to be beneficial as an augmenting strategy in treatment-resistant schizophrenia (Grover et al., 2019; Sinclair et al., 2019; Chiu et al., 2020) with maintenance ECT providing an effective form of relapse prevention (Ward et al., 2018), in mania (Elias et al., 2021), catatonia (Kellner et al., 2020), severe agitation (Grover et al., 2019), specific mental disorders in multiple sclerosis (Yahya and Khawaja, 2021), and prepartum and postpartum psychotic states (Gazdag et al., 2009). Even for children and adolescents, no absolute contra indications for ECT have been seen (Døssing and Pagsberg, 2021). Being independent of its antidepressant effects, ECT also has a clear anti-suicidal effect (Kellner et al., 2005), which suggests the use of ECT as the first choice of treatment for patients at a high risk of suicide (Fink et al., 2014).

### Therapeutic Role of Electroconvulsive Therapy in Treatment Guidelines

Electroconvulsive therapy, which is among the oldest and most controversial treatments in the field of psychiatry, had its 80th birthday 2 years ago (Bayes and Parker, 2018; Gazdag and Ungvari, 2019). Its major and widely accepted role as an essential method in the armamentarium of treatments in psychiatry has been damaged over the recent decades. The position of ECT in modern psychiatry is defined by its well-proven efficacy in most important clinical indications, including severe and treatment-resistant depression and schizophrenia, catatonia, and prepartum and postpartum affective and psychotic states in which ECT may even be lifesaving. National and international psychiatric treatment guidelines vary widely (Parker et al., 2017) but position ECT predominantly in the last stages of their treatment algorithms (Davidson, 2010; Bauer et al., 2013; Milev et al., 2016; DGPPN et al., 2017; Bayes and Parker, 2018; Grunze et al., 2018; Gaebel et al., 2019; Weiss et al., 2019). Accordingly, in many Western countries, severe and treatment-resistant depression is the main indication for ECT while the number one indication worldwide is schizophrenia (Leiknes et al., 2012; Kellner et al., 2020).

German treatment guidelines follow a rather ECT-supportive path but limit the first-line indication for ECT to delusional depression, depressive stupor, schizoaffective psychosis with severe depressive mood, major depression with high suicidality or refusal to eat, and acute life-threatening pernicious catatonia (Folkerts et al., 2003). The medical profession behind the statement has established that “refraining from ECT would mean an ethically unjustifiable restriction of the right of critically ill patients, who are often at risk for suicidal behavior, to get the best possible treatment.” In line with this position, review publications (Kellner et al., 2020) and meta-analyses suggest to advertise ECT progressively to be used in a wider indication even in patients with potential risk profiles as ECT has been proven to be effective and safe during the first trimester of pregnancy (Calaway et al., 2016) in children (Døssing and Pagsberg, 2021) as well as in old-old adults aged 80 years and older (Plakiotis et al., 2014; Kellner et al., 2016; McCall et al., 2018), and in patients with behavioral and psychological symptoms of dementia (Swierkosz-Lenart et al., 2019).

### Stigma Against Electroconvulsive Therapy

Convincing efficacy data and professional recommendations for ECT contrast with the negative media portrayal of ECT and its earlier misuse that may have contributed to its negative professional and public perceptions were indicated repeatedly in attitude surveys. This negative attitude has played an important role in the decreasing use of ECT in the developed world and a reduction in access to ECT, which constitutes a violation of psychiatric patients’ right to effective treatment (Gazdag and Ungvari, 2019). Psychiatrists’ most common barriers to referring patients to ECT were the patients’ negative attitudes and difficulty of arranging adequate social support during an ECT course (Dauenhauer et al., 2011). When introducing ECT as a new treatment, clinic staff’s attitudes toward ECT were considerably improved if formal information adapted to each profession was given, with special emphasis on nurses, and contact to ECT-experienced patients (Scholz-Hehn et al., 2019). Even proper information on ECT for the students of medicine and psychology was helpful to stimulate a positive attitude in the early stages of the health professionals’ careers (Aki et al., 2013; Aoki et al., 2016; Alexander et al., 2020a,b). Several lines of scientific evidence confirmed that a remarkably better attitude was reached by comprehensive, clear, and open information about the specific data of the ECT applying unit (Li et al., 2016).

The stigmatizing attitudes and behaviors concerning ECT are closely related to one‘s personal and factual knowledge (Kring et al., 2018). Accurate information and learning by participating in EKTs were proven to increase knowledge and improve attitudes toward ECT significantly in physicians, nurses, patients, and relatives (Aoki et al., 2016; Li et al., 2016; Alexander et al., 2020a,b). This may include watching a live ECT session by medical interns (Gazdag et al., 2009) or patients‘ relatives (Elias et al., 2019), the latter improving the patients’ satisfaction with the experience of treatment (Chakrabarti et al., 2010). In this context, patients and their relatives will always be a key factor for ECT. ECT-related anxiety is a highly prevalent phenomenon to be present in 14–75% of patients and is most often linked to worries about severe and long-lasting physical damage (Obbels et al., 2017). Both, female patients and patients with psychotic depression, experienced more ECT-related anxiety before the start of ECT (Obbels et al., 2020b). Not only the improvement of depression by ECT (Fisher, 2012; Obbels et al., 2020a) but also cognitive behavioral therapy (Wo et al., 2015) had helped significantly to overcome the anxiety related to ECT. In this context, the work of psychologists plays a key role in the overall treatment success (Fisher, 2012).

Besides, there are important facts to remember when trying to understand an extremely low ECT rate in some Western countries, and particularly in Germany. Patients may develop their fears from the unfortunate history of this form of therapy, displayed in “One flew over the cuckoo’s nest,” a movie that moved the general public, with a deep impact in the United States (Alexander et al., 2020a,b). An irrational attitude toward ECT is therefore more a rule than an exception. In Europe, the application of “electroshock” therapy during the period of National Socialism in Germany, Austria, Poland, and other countries (Rzesnitzek and Lang, 2017) with the mass killing of people with intellectual disabilities and severe psychiatric disorders (Gazdag et al., 2017b) puts ECT in the *Shadow of the Gas Chambers* (Czech et al., 2020). Respective variations of ECT utilization rate are found in central-eastern European countries with Slovenia at one end, where ECT is banned (Gazdag et al., 2017a,b). As a long-lasting consequence, the public attitude to ECT in Europe remains controversial. Studies on the general public indicate that negative attitudes are rooted in individuals’ moral and ethical objections to ECT, particularly the emotional components of such attitudes (Alexander et al., 2020b).

All of these data point out the need to generate individual experience and to collect safety data for each particular ECT facility—as done in this study—to be able to provide appropriate information to both patients and medical staff.

## Conclusion

The present analysis of 3 years of ECT in a psychiatric hospital revealed an extremely low incidence rate of 0.097% of pLTAEs that required medical action. It translates to a simpler expression that roughly one of 1,000 treatments in this hospital possibly endanger the patient and that medical action safely eliminates this danger. This may drive the conclusion that ECT is a rather safe treatment when performed in a controlled setting. Objections to ECT seem to be driven more by stigma than by evidence-based risk for physical harm. ECT requires a controlled setting with access to intensive care methods and the presence of an experienced physician applying ECT, a well-trained nurse, and an anesthetist for proper anesthesia and emergency intervention. The beneficial risk profile of ECT performed in the standard care of psychiatric hospitals suggests a more generous indication of this treatment method. We recommend that ECT facilities collect their own safety data to allow a reliable judgment of their institutional ECT risk profile.

## Data Availability Statement

The original contributions presented in the study are included in the article/supplementary material, further inquiries can be directed to the corresponding author/s.

## Ethics Statement

The studies involving human participants were reviewed and approved by Ethikrat Otto-Friedrich-Universität Bamberg. The patients/participants provided their written informed consent to participate in this study. Written informed consent was obtained from the individuals for the publication of any potentially identifiable images or data included in this article.

## Author Contributions

VH performed data analysis derived from safety protocols and patient documents, analyzed the three reported cases with severe AEs, and contributed to the writing of the manuscript. GH created the study protocol, co-analyzed the three reported cases with severe AEs, double-checked data analysis, and contributed to the writing of the manuscript. CZ organized ECT and double-checked the case report and safety protocol analyses. SG double-checked the data analysis and interpretation of data and contributed to the writing of the manuscript. WT controlled data quality, performed the statistical analysis, and contributed to the writing of the manuscript. All authors contributed to the final manuscript and submission.

## Conflict of Interest

The authors declare that the research was conducted in the absence of any commercial or financial relationships that could be construed as a potential conflict of interest.

## Publisher’s Note

All claims expressed in this article are solely those of the authors and do not necessarily represent those of their affiliated organizations, or those of the publisher, the editors and the reviewers. Any product that may be evaluated in this article, or claim that may be made by its manufacturer, is not guaranteed or endorsed by the publisher.
